# Routine Extubation in the Operating Room After Minimally Invasive Aortic Valve Replacement †

**DOI:** 10.3390/jcm14103401

**Published:** 2025-05-13

**Authors:** Mihee Lim, Minho Ju, Chee-Hoon Lee, Younju Rhee, Hye-Jin Kim, Jung-Pil Yoon, Hong-Sik Shon, Hyung Gon Je

**Affiliations:** 1 Department of Cardiovascular and Thoracic Surgery, Research Institute for Convergence of Biomedical Science and Technology, Pusan National University Yangsan Hospital, Pusan National University College of Medicine, Yangsan 50612, Republic of Korea; smmh1008@gmail.com (M.L.); 2 Department of Anesthesia and Pain Medicine, Pusan National University Yangsan Hospital, Pusan National University College of Medicine, Yangsan 50612, Republic of Korea; wizdumb@naver.com (J.-P.Y.); 3 Department of Cardiovascular and Thoracic Surgery, Research Institute for Convergence of Biomedical Science and Technology, Seoul National University Bundang Hospital, Seoul National University College of Medicine, Seongnam 13620, Republic of Korea

**Keywords:** aortic valve replacement, on-table extubation, right anterior mini-thoracotomy

## Abstract

**Objective**: The present study aimed to evaluate the feasibility and safety of performing extubation in the operating room following aortic valve replacement (AVR) via right anterior mini-thoracotomy (RAMT), as the safety profile of this approach has not been fully established. **Methods**: We conducted a retrospective analysis of patients who underwent isolated AVR through a RAMT between February 2012 and December 2023. Emergency cases and reoperations were excluded. Patients were categorized according to the location of extubation—either in the operating room (on-table) or in the intensive care unit (ICU). Multivariable logistic regression analysis was used to identify predictors associated with successful on-table extubation. **Results**: Among 423 patients who underwent non-emergent isolated AVR, 73.3% were extubated in the operating room. This group was characterized by younger age, lower EuroSCORE II, and higher preoperative serum albumin levels. While the surgical techniques did not differ between groups, those extubated on-table had significantly shorter cardiopulmonary bypass times (84.0 [68.0–104.0] vs. 104.0 [85.0–131.5], *p* < 0.001). Although early postoperative outcomes were comparable, the on-table extubation group had significantly shorter ICU stays (24.0 [22.0–26.0] vs. 25.0 [23.0–30.0], *p* < 0.001) and hospital stays (5.0 [4.0–6.0] vs. 6.0 [5.0–8.0], *p* < 0.001). A predictive model incorporating age, albumin levels, and cardiopulmonary bypass time demonstrated a predictive accuracy of approximately 78.4% for on-table extubation success. **Conclusions**: Extubation in the operating room was found to be both safe and effective for the majority of patients undergoing isolated AVR via RAMT. It was associated with low reintubation rates and significantly reduced lengths of ICU and hospital stays. These findings support the adoption of routine on-table extubation in suitable patients undergoing this procedure.

## 1. Introduction

The trend in cardiac surgery is increasingly moving towards less invasive procedures, driven by advancements in surgical technology and improved patient recovery outcomes [1,2,3]. This shift includes a significant transition from full sternotomy to right anterior mini-thoracotomy (RAMT) for aortic valve replacement (AVR), which minimizes surgical trauma and enhances patient recovery per Enhanced Recovery After Cardiac Surgery (ERCAS) protocols [4,5,6]. These protocols emphasize shorter mechanical ventilation times and reduced blood transfusion needs, highlighting the clinical benefits of modern surgical approaches.

Early extubation in the intensive care unit (ICU) within 6 h post operation has been associated with improved outcomes, including shorter hospital stays and reduced costs, without increasing the risks of complications such as reintubation or pulmonary issues [7]. Moreover, on-table extubation, already safely implemented in other cardiac surgeries, such as off-pump coronary artery bypass (OPCAB) graft, coronary artery bypass graft (CABG), and minimally invasive mitral valve surgery, has demonstrated similar benefits [8,9]. Despite these benefits, the applicability of on-table extubation immediately after cardiopulmonary bypass (CPB) in AVR remains underexplored [10]. This uncertainty is significant given the higher risk of postoperative bleeding associated with aortotomy in AVR procedures. Conventional wisdom has also advocated for a stable postoperative period post CPB to mitigate risks such as stroke, seizure, or arrhythmias, historically limiting immediate extubation in the operating room (OR) [11].

This study compares perioperative outcomes between on-table and ICU extubation in patients undergoing non-emergency isolated AVR through RAMT. We hypothesize that routine on-table extubation conducted under controlled conditions is safe and feasible for these patients without increasing perioperative morbidity or early mortality. Our study seeks to bridge the gap in current practice by offering a deeper understanding of the potential benefits and limitations of on-table extubation in AVR.

## 2. Materials and Methods

### 2.1. Ethical Statement

The Pusan National University Yangsan Hospital Institutional Review Board approved this study (approved 29 March 2024, approval 55-2024-033). Informed consent was waived owing to the retrospective study design and the use of deidentified data.

### 2.2. Study Population

**Figure 1:**
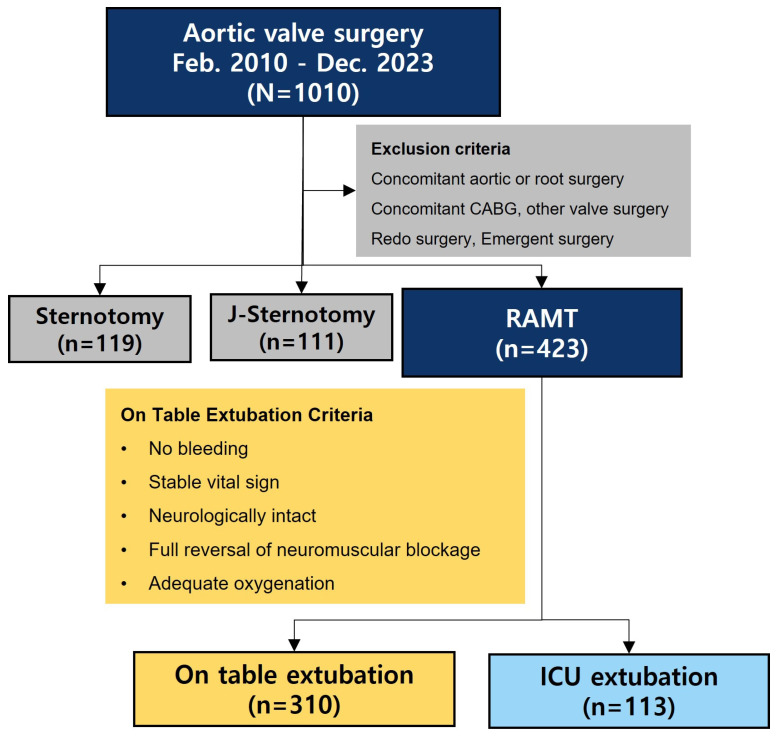
Patient flow diagram. RAMT—right anterior mini-thoracotomy; ICU—intensive care unit.

This retrospective study used prospectively collected data from our institutional database. Between February 2012 and December 2023, 1010 consecutive patients underwent AVR. To focus on isolated primary non-emergent AVR, we excluded concomitant aortic or root surgery, CABG, aortic valve valvuloplasty, other valve surgery, and redo and emergent surgery (Figure 1).

### 2.3. Surgical Techniques

All patients were intubated with a single-lumen endotracheal tube, and transesophageal echocardiography was performed. Patients underwent one-lung ventilation during surgery by inserting an endo-bronchial blocker (EZ-Blocker TM; Teleflex Medical OEM, Plymouth, MN, USA) into the endotracheal tube. Cannulation for CPB was performed using the femoral artery and vein.

Surgical access was achieved through a horizontal 6 cm skin incision, with lower rib cutting performed at the second or third intercostal space. During the incision, preemptive intercostal nerve blocks were administered with ropivacaine. To alleviate patient discomfort, no additional port for the camera scope was made. Instead of chest tubes, two Jackson–Pratt drains were placed, reusing the site previously occupied by the aortic cross-clamp (ACC) during surgery. To maintain continuous local anesthesia, a pain infusion pump was inserted during wound closure to deliver ropivacaine gradually to the pleura and muscle layer for 3 days.

### 2.4. Anesthesia Strategy for Routine On-Table Extubation

Anesthesia included the use of propofol (1.0–2.0 mg/kg) for sedation, rocuronium for muscle relaxation (0.8 mg/kg), and a small dose of remifentanil. Anesthesia was maintained using a short-acting volatile agent (sevoflurane) and a small amount of remifentanil (1–3 mL/h). The infusion of rocuronium (10 mg/h) was typically stopped when releasing the ACC, preparing the patient to breathe spontaneously by the time of skin suture completion. Once the decision for on-table extubation was made, Sugammadex (Bridion, Merck & Co., Inc., Rahway, NJ, USA), a neuromuscular blockade reversal agent, was administered immediately after skin closure. To ensure complete recovery of neuromuscular function prior to extubation, we utilized the TwitchView^®^ quantitative neuromuscular monitor (Blink Device Company, Seattle, WA, USA) in all cases. This EMG-based device measures the electrical response of muscles to peripheral nerve stimulation and provides an objective assessment of neuromuscular transmission, including the train-of-four (TOF) ratio. Extubation was performed only after confirming a TOF ratio > 0.9, in accordance with standard safety guidelines.

### 2.5. On-Table Extubation Criteria

On-table extubation has been implemented in isolated AVR patients since 2012, and the rate of on-table extubation has gradually increased throughout the study period. After confirming a minimal reintubation rate, we implemented a strategy considering all RAMT-isolated AVR patients as candidates for on-table extubation. After a thorough discussion between anesthesiologists and surgeons regarding hemodynamic instability after CPB termination and the potential for bleeding at the surgical site due to coagulopathy, on-table extubation was chosen. Adequate spontaneous respiratory function was assessed by ensuring a tidal volume greater than 5 mL/kg and a respiratory rate between 10 and 25 breaths per minute. Patients were required to be hemodynamically stable, with low-dose inotropic or vasopressor support permitted (i.e., dobutamine ≤ 5 μg/kg/min and norepinephrine ≤ 0.1 μg/kg/min). Additional criteria included oxygen saturation above 95% with a fraction of inspired oxygen (FiO_2_) of 0.4 or less, normothermia (core temperature > 36 °C), and the return of consciousness along with protective airway reflexes, such as cough and gag reflexes. These conditions were consistently evaluated prior to extubation.

Given the variability in preoperative hemoglobin (Hb) levels among patients, no absolute threshold was set for Hb, and extubation proceeded even if Hb levels were low, provided no surgical bleeding occurred. Extubation was withheld if patients exhibited irregular rhythms (e.g., atrioventricular block) postoperatively or required significant inotropic support, indicating hemodynamic instability. Standard protocols dictate the assessment of ventilatory status and the fulfillment of extubation criteria.

### 2.6. Postoperative Pain Management

At the end of surgery, all patients received a single intravenous injection of acetaminophen (1 g). In addition, a bolus dose of a custom analgesic mixture containing fentanyl and nefopam hydrochloride (AcuPain^®^ (Beacon Pharmaceuticals PLC, Dhaka, Bangladesh)) was administered intravenously. The fentanyl dose was weight-based, at 1 mcg/kg, and was given to initiate postoperative analgesia and prepare for intravenous patient-controlled analgesia (IV-PCA). This protocol was applied uniformly to patients who underwent on-table extubation.

In contrast, patients who were extubated in the intensive care unit (ICU) did not receive an additional bolus dose at the end of surgery. Instead, they were transferred to the ICU with a continuous infusion of remifentanil (Ultiva^®^ (Morgantown, WV, USA)), maintained from the intraoperative anesthesia regimen.

### 2.7. ERCAS

Extubated patients drank water orally after 2 h and ate porridge after 6 h. The patients were allowed to see their family members within 1 h after surgery for emotional support. Extubated patients spent their time sitting in bed and were taught to use an inspirometer or blow up a balloon. ICU nurses taught active sputum clearance to patients. Patients with stable hemodynamics were routinely transferred to the general ward the next day and began early ambulation.

### 2.8. Study Endpoint

The primary outcomes were the need for reintubation and the length of ICU and hospital stay. The secondary outcomes were in-hospital surgery-related morbidities and survival. An analysis of risk factors influencing on-table extubation was conducted. Postoperative morbidities were defined using standard Society of Thoracic Surgeons data element definitions. Early mortality was defined as death within 30 days of surgery. Patients’ survival and death dates were confirmed through the National Health Insurance data.

### 2.9. Statistical Analysis

Variables are expressed as frequency and percentage for categorical data and mean ± standard deviation or median with interquartile range (IQR) for numeric data. Group differences were tested using the chi-square or Fisher’s exact test for categorical data and the independent *t*-test or Mann–Whitney *U* test for numeric data as appropriate. We used Shapiro–Wilk’s test to check for normal distribution.

Univariate and multivariate analyses, using binary logistic regression, were performed to identify prognostic factors independently related to outcome. Nomogram development began by identifying patient characteristics that were predictive of outcomes in the multivariate logistic regression model. Overall survival probability was estimated using the Kaplan–Meier method. The receiver operating characteristic curve assessed the sensitivity and specificity of the scoring system used to predict patient outcomes.

All statistical analyses were performed using SPSS 26.0 (IBM Corp. Released 2019. IBM SPSS Statistics for Windows, Version 26.0. Armonk, NY, USA: IBM Corp), R 4.1.2 (R Core Team [2021], R Foundation for Statistical Computing, Vienna, Austria. URL https://www.R-project.org/), and MedCalc Statistical Software version 19.2.6 (MedCalc Software Ltd., Ostend, Belgium; https://www.medcalc.org; 2020). *p* < 0.05 was considered statistically significant. The standardized mean differences (SMDs) of 0.2, 0.5, and 0.8 are considered small, medium, and large, respectively.

## 3. Results

### 3.1. Patients’ Characteristics

Over the study period, 423 patients underwent nonemergent isolated AVR via RAMT: 310 (73.3%) underwent on-table extubation and 113 (26.7%) underwent ICU extubation. The incidence of on-table extubation increased over time, with a steep inflection point in 2019 (Figure S1). Baseline patient characteristics are presented in Table 1.

Surgery due to aortic valve stenosis accounted for 79.4% of the on-table extubation group and 81.4% in the ICU extubation group, with no significant difference in the concomitant surgical ablation (3.2% and 4.4%, respectively). The ICU extubation group had a higher prevalence of tissue valves and a slightly higher use of sutureless and rapid deployment (SURD) aortic valves. The on-table extubation group had shorter CPB times and ACC times (Table 1).

### 3.2. Early Clinical Outcomes

Two early deaths (0.5%) occurred without significant differences between the two groups (*p* < 0.463). The lengths of ICU and hospital stays were shorter in the on-table extubation group (*p* < 0.001; Table 2). The primary outcome, reintubation after on-table extubation incidence, was higher in the ICU extubation group. Postoperative tube drain, the rate of transfusion requirement, and the need for reoperation due to bleeding were statistically higher in the ICU extubation group. The tendency for bleeding-prone patients to not undergo on-table extubation may be attributed to the decision-making process of the attending physician. The observed trend of bleeding in the OR does not appear to significantly impact subsequent outcomes, regardless of early ventilator removal. Factors such as rhythm disturbances, including temporary AV block, also differed between the two groups, influencing the decision for extubation.

**Figure 2:**
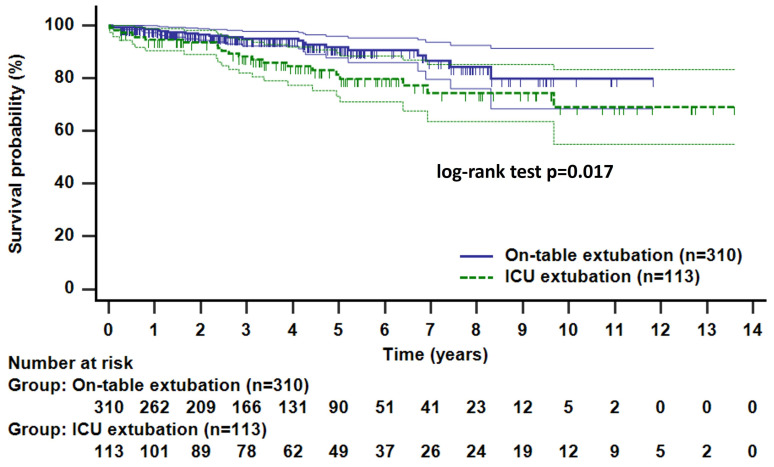
Kaplan–Meier curves of overall survival.

Postoperative low cardiac output syndrome and atrial fibrillation occurred more frequently in the ICU extubation group, likely due to the higher proportion of older patients and those with a higher EuroSCORE Ⅱ in the ICU extubation group. The survival curves differed between the two groups for the same reasons (Figure 2). However, the rates of home discharge post surgery were similar in both groups, and the readmission rate within 1-month post discharge was comparable.

### 3.3. Prognostic Index Based on Successful On-Table Extubation

**Figure 3:**
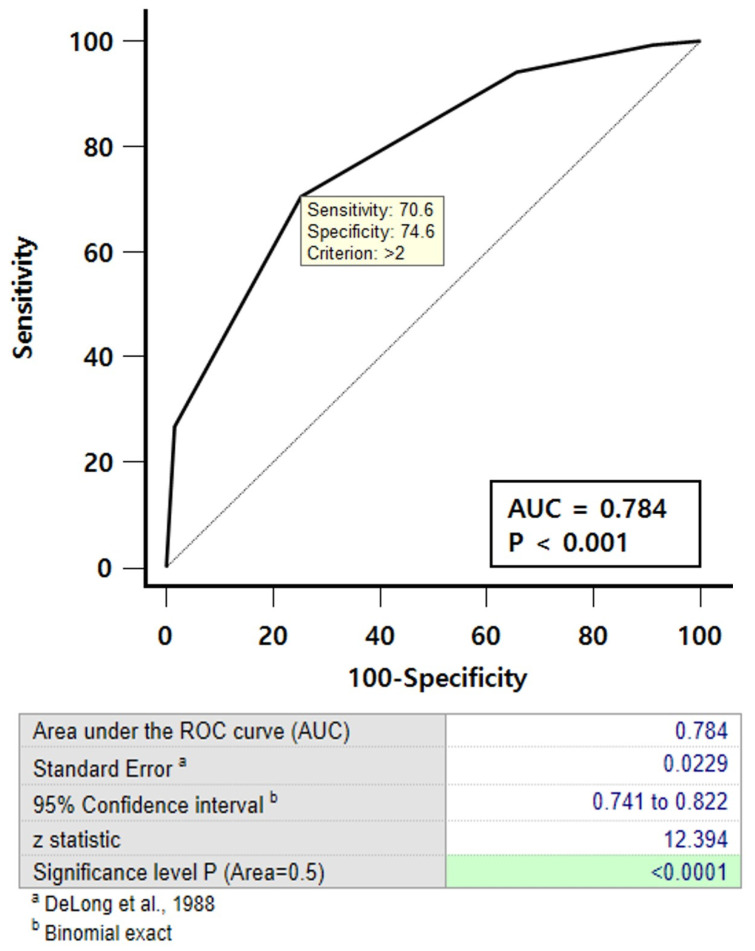
The receiver operating characteristic (ROC) curve of the risk prediction model for on-table extubation was constructed. Model discrimination was assessed by computing the area under the ROC curve (area under curve c-index), serving as an indicator of the model’s ability to distinguish between patients who succeeded and failed in on-table extubation. Comparison of ROC curves using the DeLong method [13].

Among the 310 patients who underwent on-table extubation, excluding one who required reintubation, we conducted an analysis to identify prognostic factors for successful on-table extubation. We used a logistic regression model (LRM) and recursive partitioning analysis (RPA) to develop a new prognostic scoring system. Significant factors from the multivariate logistic regression—age, preoperative serum albumin level, and duration of CPB time—were included to develop the nomogram (Table 3, Figure S2). The nomogram is based on proportionally converting each regression coefficient in the multivariate LRM to a scale from 0 to 100 points; the variable with the highest β coefficient (absolute value), CPB time, was assigned 100 points. The associated prediction factors of on-table extubation in the multivariate model were also categorized according to clinical cut-points to create the score model. The scoring system was constructed using regression coefficients multiplied by 0.5 and rounded to the nearest integer to calculate the weights [12]. The simple prognostic scoring system was developed based on the prognostic scores and regression coefficients in multivariate logistic analysis and RPA (Table 4). A cumulative score of ≥3 allows for safe on-table extubation with a predictive accuracy of 78.4% (Figure 3).

## 4. Discussion

This study compared perioperative outcomes after on-table and ICU extubation in 423 patients who underwent non-emergency, isolated AVR over 13 years. Initially, on-table extubation was applied for selected patients. However, by the final year of the study, most patients undergoing nonemergency, isolated AVR underwent on-table extubation (96.6% in 2023).

On-table and ICU extubation did not differ in the incidence of reintubation, postoperative acute renal failure, postoperative stroke, or 30-day mortality. On-table extubated patients had a lower incidence of prolonged ventilation, fewer ICU hours, and a shorter postoperative hospital length of stay, demonstrating the safety of this strategy. Conversely, postoperative bleeding, transfusion requirements, and the need for reoperation due to bleeding were higher in the ICU extubation group. A formal cost analysis was not performed; however, a previous study suggested that on-table extubation is a cost-effective strategy [14], with patients in this group having shorter ICU and hospital lengths of stay.

The concept of pursuing on-table extubation is not new in cardiac surgery, as several studies have already underscored the benefits associated with a fast-track extubation, especially in OPCAB grafting [15], CABG, and minimally invasive cardiac surgery [16]. Research on on-table extubation following AVR is scarce and limited [10]. Previous reports have indicated the safety of on-table extubation after AVR in patients who underwent additional epidural anesthesia for pain control. However, post-operative pain may make it challenging to control blood pressure, increasing the risk of bleeding from the aortic incision site, which can lead to excessive bleeding and tamponade.

Due to previous successful efforts, on-table extubation was extended to most patients, resulting in all early patients recovering well, without failure. Collaboration with anesthesia is paramount; sevoflurane, an ultrafast-acting anesthesia, is typically used for anesthesia maintenance, while the anesthesia team routinely suspends rocuronium administration as the surgical team prepares to release the ACC. Sugammadex (Bridion, MERCK Connect, Rahway, NJ, USA), the rocuronium antagonist, is given when the patient awakes. Once neuromuscular blockade has fully recovered and the patient’s consciousness, cough, and gag reflex have returned, on-table extubation is performed. Intravenous analgesics are administered for adequate pain control. Additionally, to minimize pain, the surgical team administers ropivacaine at the start of surgery to perform preemptive intercostal nerve blocks. Special attention is paid to preventing rib fractures, and two thin Jackson–Pratt drains are inserted. These concerted efforts to alleviate pain have ensured the safe implementation of routine on-table extubation.

Patients could be maintained stably after on-table extubation because the CPB and ACC times have significantly decreased compared to the past [17,18] due to factors such as the surgeon’s mastery of the learning curve associated with RAMT for AVR [19,20]. Additionally, advancements in surgical techniques, such as the SURD aortic valve and the Cor-Knot (LSI Solutions, Victor, NY, USA) automated fastener, have further reduced surgical times, making them comparable to those of conventional sternotomy [21,22].

Using our data, we developed an algorithm to predict the likelihood of successful on-table extubation following isolated AVR via RAMT. This algorithm, derived from univariable and multivariable logistic regression analyses, incorporates preoperative and operative factors, including age, sex, chronic lung disease, dialysis, preoperative serum Hb, serum albumin, CPB time, NYHA classification, and left ventricular ejection fraction (Figure S2). The proposed algorithm (Table 4) suggests that in centers where on-table extubation is not routinely performed after AVR, patients < 70 years old with preoperative serum albumin levels > 4.0 (g/dL), adequate nutritional status, and shorter CPB times (<90 min) may safely undergo on-table extubation.

Patients who undergo cardiac surgery often cite the process of waking up from anesthesia and weaning off the ventilator as exceedingly challenging [23]. During the gradual reduction in ventilator support, patients must be roused from consciousness with their hands restrained to avoid unintended extubation [24]. While a shorter duration of this process is desirable, it may vary depending on hospital protocols and medical considerations. The primary goal of all cardiac surgeries is restoring cardiac function; however, patients universally aspire to recover as quickly as possible with minimal invasiveness, aiming to return to their daily lives expeditiously. Reducing discomfort and additional damage during cardiac surgery and postoperative care is crucial for improving patient experience [25]. Modern surgical techniques, such as RAMT, and evidence-based early recovery protocols, such as on-table extubation, can effectively achieve this goal while ensuring satisfactory outcomes.

### Limitations

This study has several limitations. First, this was an observational study, with the inherent limitations of a non-randomized trial. Second, the retrospective nature of this study makes it impossible to entirely exclude the effects of intraoperative confounding factors that cannot be easily quantified objectively, although the preoperative demographic characteristics of the ICU extubation group and the on-table extubation group are comparable. Nevertheless, a strength of this study is that the surgical and anesthesia protocols were maintained relatively consistently throughout the study period. Unfortunately, we do not have data on patients who were initially scheduled for on-table extubation but eventually required ICU extubation.

## 5. Conclusions

On-table extubation carried minimal risk of reintubation and was linked to shorter ICU and hospital stays for most patients who underwent isolated AVR through RAMT. These results advocate for the broader implementation of routine on-table extubation for isolated AVR through RAMT.

## Figures and Tables

**Table 1:** Preoperative patient characteristics and operative profile.

		Extubation			
Variable	Overall (*n* = 423)	Yes (*n* = 310)	No (*n* = 113)	*p*	SMD
Age, years	66.9 ± 10.8	65.5 ± 10.9	70.8 ± 9.5	<0.001	−0.52
Female	162 (38.3)	109 (35.2)	53 (46.9)	0.028	0.24
BSA	1.7 [1.5–1.8]	1.7 [1.6–1.9]	1.6 [1.5–1.8]	<0.001	0.44
HTN	310 (73.3)	228 (73.5)	82 (72.6)	0.840	0.02
DM	109 (25.8)	84 (27.1)	25 (22.1)	0.301	0.12
CLD	24 (5.7)	17 (5.5)	7 (6.2)	0.780	−0.03
CKD					
1	98 (23.2)	83 (26.8)	15 (13.3)	0.001	0.34
2	159 (37.6)	121 (39.0)	38 (33.6)		0.11
3	134 (31.7)	89 (28.7)	45 (39.8)		−0.24
4	20 (4.7)	11 (3.5)	9 (8.0)		−0.19
5	12 (2.8)	6 (1.9)	6 (5.3)		−0.18
Dyslipidemia	330 (78.0)	245 (79.0)	85 (75.2)	0.402	0.09
Hemoglobin	12.7 [11.1–14.0]	13.0 [11.3–14.1]	12.3 [10.6–13.5]	0.007	0.32
Platelet	206.0 [164.0–245.0]	206.0 [168.8–242.0]	203.0 [157.5–248.5]	0.277	0.05
Albumin	4.2 [3.9–4.4]	4.2 [4.0–4.5]	4.0 [3.7–4.3]	<0.001	0.60
BNP	184.0 [65.0–525.0]	149.0 [51.0–413.3]	410.0 [135.5–1130.5]	<0.001	−0.47
CVA	43 (10.2)	26 (8.4)	17 (15.0)	0.135	−0.21
NYHA 3/4	174 (41.1)	118 (38.1)	56 (49.6)	0.034	−0.23
Urgent	60 (14.2)	32 (10.3)	28 (24.8)	<0.001	0.39
EuroSCORE II	1.6 [1.0–3.0]	1.4 [0.9–2.3]	2.3 [1.2–5.0]	<0.001	−0.41
AF	21 (5.0)	16 (5.2)	5 (4.4)	0.758	0.03
EF	62.0 [56.0–66.0]	62.0 [56.0–67.0]	60.0 [54.0–66.0]	0.072	0.17
Operative profile					
Isolated AVR	397 (93.9)	295 (95.2)	102 (90.3)	0.064	0.19
Sternotomy conversion	7 (1.7)	1 (0.3)	6 (5.8)	0.002	−0.32
SURD valve	115 (27.2)	79 (25.5)	36 (31.9)	0.192	−0.14
Ablation	15 (3.5)	10 (3.2)	5 (4.4)	0.558	−0.06
CPB time					
median [IQR]	90.0 [72.0–111.0]	84.0 [68.0–104.0]	104.0 [85.0–131.5]	<0.001	−0.77
Cross clamp time					
median [IQR]	65.0 [53.0–84.0]	63.0 [51.0–78.0]	79.0 [57.5–98.0]	<0.001	−0.63

SMD—standardized mean difference; BSA—body surface area; BNP—B-type natriuretic peptide; HTN—hypertension; DM—diabetes mellitus; CLD—chronic lung disease; CKD—chronic kidney disease; PNB; CVA—cerebrovascular accident; NYHA—New York Heart Association; EuroSCORE II—European System for Cardiac Operative Risk Evaluation II; AF—atrial fibrillation; EF—ejection fraction; ASR—aortic steno-regurgitation; AVR—aortic valve replacement; SURD—sutureless and rapid deployment; CPB—cardiopulmonary bypass; IQR—interquartile range; SD—standard deviation. Data are presented as mean ± SD, median [IQR], or number (%).

**Table 2:** Early clinical outcomes.

		Extubation		
Variable	Overall (*n* = 423)	Yes (*n* = 310)	No (*n* = 113)	*p*
Hospital stay (days)	5.0 [4.0–7.0]	5.0 [4.0–6.0]	6.0 [5.0–8.0]	<0.001
Intensive care unit stay (h)	24.0 [22.0–26.0]	24.0 [22.0–26.0]	25.0 [23.0–30.0]	<0.001
LCOS	43 (10.2)	25 (8.1)	18 (15.9)	0.018
Atrial fibrillation	98 (23.2)	62 (20.0)	36 (31.9)	0.011
Atrioventricular block	16 (3.8)	4 (1.3)	12 (10.6)	<0.001
Pacemaker insertion	5 (1.2)	3 (1.0)	2 (1.8)	0.613
Mechanical ventilation time (h)	0.0 [0.0–2.0]	0.0 [0.0–0.0]	5.0 [3.0–10.0]	<0.001
Prolonged vent (≥72 h)	4 (0.9)	1 (0.3)	3 (2.7)	0.060
Pneumonia	3 (0.7)	1 (0.3)	2 (1.8)	0.175
Reintubation	4 (0.9)	1 (0.3)	3 (2.7)	0.060
New onset dialysis	2 (0.5)	2 (0.6)	0 (0.0)	1.000
Stroke	1 (0.2)	0 (0.0)	1 (0.9)	0.267
Delirium	8 (1.9)	4 (1.3)	4 (3.5)	0.218
Bleeding amount	302.8 ± 254.0	273.2 ± 197.5	383.8 ± 355.6	0.054
Bleeding reoperation	15 (3.5)	7 (2.3)	8 (7.1)	0.032
Transfusion	115 (27.2)	50 (16.1)	65 (57.5)	<0.001
30-Day mortality	2 (0.5)	1 (0.3)	1 (0.9)	0.463
Readmission (<30 days)	25 (5.9)	19 (6.1)	6 (5.3)	0.752

LCOS—low cardiac output syndrome. Data are presented as mean ± SD, median [IQR], or number (%).

**Table 3:** Univariate and multivariate logistic regression analysis for successful on-table extubation.

	Univariate Analysis			Multivariate Analysis ^1^		
Variables	OR	95% CI	*p*-Value	OR	95% CI	*p*-Value
Age, years	0.95	(0.92–0.97)	<0.001	0.93	(0.90–0.96)	<0.001
CLD (yes vs. no)	0.72	(0.30–1.74)	0.470			
Dialysis (yes vs. no)	0.36	(0.11–1.13)	0.079			
Hemoglobin	1.18	(1.06–1.32)	0.004			
Albumin	4.19	(2.43–7.22)	<0.001	3.32	(1.78–6.19)	<0.001
NYHA (yes vs. no)	0.61	(0.40–0.94)	0.025			
Urgent (urgent vs. elective)	0.35	(0.20–0.62)	<0.001			
Ejection fraction	1.02	(0.99–1.04)	0.146			
CPB time	0.98	(0.97–0.98)	<0.001	0.97	(0.96–0.98)	<0.001
Cross clamp time	0.97	(0.97–0.98)	<0.001			

OR—odds ratio; CI—confidence interval; CLD—chronic lung disease; NYHA—New York Heart Association; CPB—cardiopulmonary bypass. ^1^ The effect of independent variables on response variables was analyzed using multivariate logistic regression. The multivariate model was created using a backward elimination method, with a removal probability of 0.05.

**Table 4:** Prognostic index based on the presence of predictors for successful on-table extubation.

		Point Contribution		
Variables		0	1	2
Age, years		≥70	<70	
Albumin		<4.0	≥4.0	
Cardiopulmonary bypass time		≥120	90~120	<90
Variable	Cut-point value	On-table extubation		AUC (*p*)
		Success	Fail	
Scoring system	>2	218	29	0.784 (<0.001)
	≤2	91	85	
Total		309	114	

AUC—area under curve.

## Data Availability

The data presented in this study are available on request from the corresponding author due to (specify the reason for the restriction).

## Supplementary Materials

The following supporting information can be downloaded at: https://www.mdpi.com/article/10.3390/jcm14103401/s1, Figure S1: Percentage of patients who underwent on-table and intensive care unit extubation each year; Figure S2: Nomogram using the multivariate logistic regression model consisted of three variables. Information about the nomogram, such as the point-linear predictor unit mapping and total points-risk of event mapping, can be viewed.

## Abbreviations

The following abbreviations are used in this manuscript:

ACC	Aortic cross-clamp
AVR	Aortic valve replacement
BNP	B-type natriuretic peptide
CPB	Cardiopulmonary bypass
CABG	Coronary artery bypass graft
ERCAS	Enhanced Recovery After Cardiac Surgery
ICU	Intensive care unit
OPCAB	Off pump coronary artery bypass
OR	Operating room
RAMT	Right anterior mini-thoracotomy
SURD	Sutureless and rapid deployment
