# Supplementary figures and images for: Routine Extubation in the Operating Room After Minimally Invasive Aortic Valve Replacement
Source: J Clin Med. 2025 May 13;14(10):3401. doi: 10.3390/jcm14103401 (PMC12112692; doi:10.3390/jcm14103401)

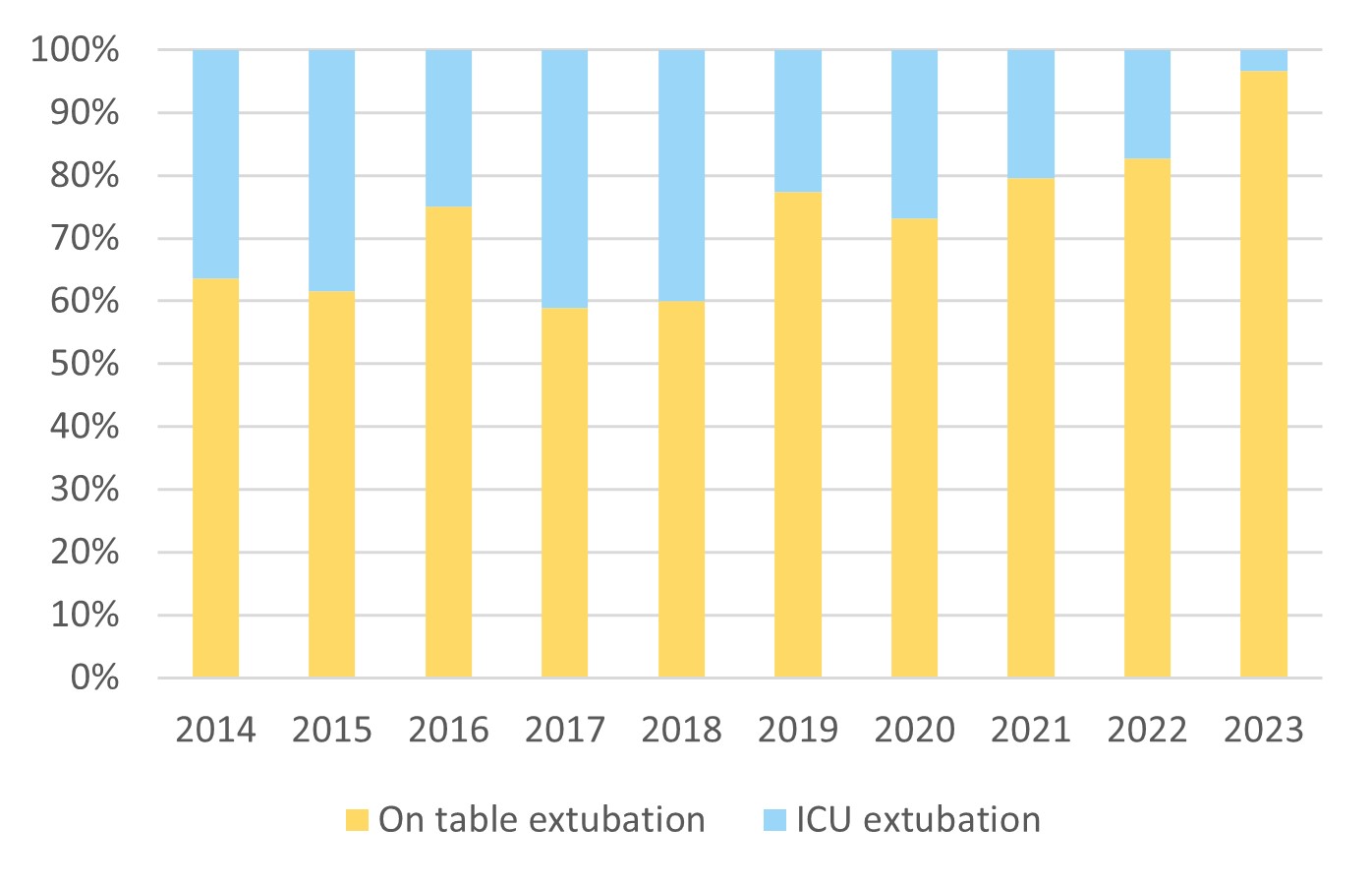

Supplement: Supplementary file 1 [file jcm-14-03401-s001.zip › Figure S1.jpg]

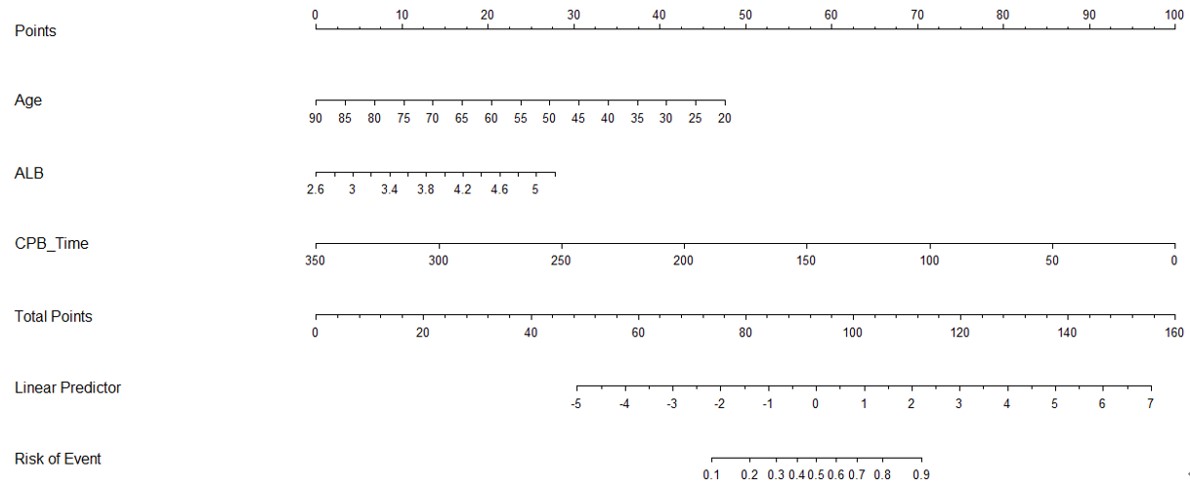

Supplement: Supplementary file 1 [file jcm-14-03401-s001.zip › Figure S2.jpg]
